# Biomechanical Analysis of Ankle Stability Following Deltoid Ligament Repair and Reconstruction

**DOI:** 10.1155/bmri/6313005

**Published:** 2025-03-25

**Authors:** Ali Ahmadi Pirshahid, Pawel Brzozowski, Olawale Sogbein, Radovan Zdero, Aaron Gee, Mansur Halai, Emil Schemitsch, David W. Sanders, Abdel Rahman Lawendy, Christopher Del Balso

**Affiliations:** ^1^Division of Orthopaedic Surgery, Department of Surgery, Western University, London, Ontario, Canada; ^2^Orthopaedic Biomechanics Lab, Victoria Hospital, London, Ontario, Canada; ^3^Division of Orthopaedic Surgery, Department of Surgery, University of Toronto, Toronto, Ontario, Canada

**Keywords:** ankle, biomechanics, deltoid ligament, reconstruction, repair

## Abstract

**Background:** The deltoid ligament has been shown to contribute to the stability of the ankle mortise, preventing valgus talar tilt, external rotation (ER), and translation. Research to date assessing the appropriateness of deltoid repair to reintroduce medial stability to a fractured ankle is unclear. Deltoid ligament reconstruction using autograft or allograft has been proposed in cases of chronic deltoid insufficiency. This biomechanical study sought to assess the stability offered by deltoid repair and reconstruction compared to the native deltoid ligament in its intact and defunctioned states.

**Materials and Methods:** Twelve (six pairs) fresh frozen cadaveric lower extremities with intact deltoid ligaments underwent biomechanical assessment in a custom-made multiaxial testing apparatus. Each specimen was tested in plantarflexion/dorsiflexion (PF/DF), inversion/eversion (IV/EV), and internal rotation (IR)/ER and analyzed for angular range of motion (ROM) and stiffness. The specimens were tested with an intact deltoid ligament and following disruption via transection. Subsequently, paired specimens were randomized to either deltoid repair or reconstruction. A single double-loaded suture anchor was used to repair the deep and superficial deltoid ligaments. The tibialis anterior tendon was used as an autograft in the reconstruction group.

**Results:** Normalized ROM and stiffness were significantly different in deficient specimens compared to all other groups during PF/DF. During IR/ER, the deficient ankle was significantly different from the intact and repair state. For IV/EV, deficient specimen ROM was significantly greater than the intact or repair states, while the stiffness for the deficient ankles was significantly less.

**Conclusion:** Deltoid repair and reconstruction were comparable in returning the ankles to an intact state and conferring stability. These results suggest that in cases with insufficient or unstable deltoid ligament where repair is not possible, reconstruction has the potential to be a reliable alternative, but further studies are warranted to understand all advantages/disadvantages.

## 1. Introduction

The ankle joint is stabilized medially and laterally by ligaments providing it with static and dynamic restraint. The deltoid ligament is the fan-shaped medial ligament, itself composed of a superficial and deep portion [1, 2]. The superficial deltoid courses from the anterior colliculus to the navicular, calcaneus, and talus. The deep deltoid attaches to the intercollicular groove and posterior colliculus proximally and inserts onto the talus [2, 3]. Numerous biomechanics studies have shown that the deltoid complex prevents valgus talar tilt, talar external rotation (ER), and translation [4–7].

Ankle fractures commonly involve disruption of ankle ligaments in addition to bony injury. When syndesmotic involvement is suspected clinically and radiographically, fixation of the syndesmosis is often carried out [8]. Likewise, a torn deltoid ligament is suspected clinically and confirmed with increased medial clear space on an x-ray. However, repairing the deltoid ligament in the acute setting remains controversial [9]. Several studies show a correlation between deltoid repair and better radiographic outcomes in early follow-up [9, 10]. A long-term study assessing the correlation between radiographic and functional outcomes in operative ankle fractures with a minimum 10-year follow-up did show a positive trend between good early radiographic outcomes and positive long-term clinical results [11]. Furthermore, a biomechanical study assessing the tibiotalar contact area after sectioning the deltoid ligament showed a significant decrease in the tibiotalar contact area medially which theoretically increases the risk of osteoarthritis [6]. This radiographic advantage does seem to translate to better postoperative pain scores, but two recent meta-analyses show conflicting results on whether it also translates to better functional scores [9, 12].

In the chronic setting, deltoid ligament insufficiency is typically seen in postinjury settings or adult-acquired flat-foot deformities. In the setting of ankle arthroplasty, valgus deformity of the ankle can increase implant edge loading and wear [13, 14]. Lack of deltoid support has therefore been cited as a contraindication to ankle arthroplasty, with one study finding a 75% change in coronal alignment within 2 years if pre-existing valgus deformity was greater than 15° [15, 16]. Chronic ankle instability has likewise been linked to a high incidence of chronic deltoid ligament insufficiency [17]. Reconstructing the deltoid ligament to correct this deformity has been gaining traction recently as ankle arthroplasty becomes an increasingly performed treatment option for ankle arthrosis [18]. However, reconstruction is often a lengthier and more involved procedure requiring harvesting and preparation of an allograft or autograft. Therefore, additional concerns with healing time, efficacy, cost-effectiveness, infection rates, and more must be considered before proceeding with this procedure.

This biomechanical study sought to assess differences in the stability of cadaveric ankle specimens in intact and deltoid deficient states. Following subsequent randomization to either deltoid repair or reconstruction, specimens were re-evaluated to test our hypothesis that deltoid repair and reconstruction provide equivalent ankle stability to that of an intact deltoid ligament.

## 2. Materials and Methods

### 2.1. Specimen Preparation and Graft Harvesting

Six pairs (12) of cadaveric specimens of the human ankle cut at the midtibia were ethically sourced (United Tissue Network, St. Petersburg, Florida, United States) and stored frozen. The ages of the specimens ranged from 58 to 83 years. Three pairs of specimens were male and three pairs were female. The specimens were thawed at room temperature for 24 h before testing. Each limb from specimen pairs was randomized to either the deltoid repair or reconstruction group. Once thawed, specimens were prepared with skin resection to allow for access to the tibialis anterior tendon (TA) and deltoid ligament. TA was harvested from each specimen prior to testing. For specimens randomized to the reconstruction group, the graft was tabularized on both ends using the Krakow suture technique and placed in moist saline. At the proximal 5 cm of each specimen, all soft tissue was cleared off the proximal tibia and fibula to allow for potting specimens in cement. Screw fixation between the fibula and tibia proximally was performed to preserve the interosseous space. The specimen was then potted in a metal box with anchoring cement (Flowstone, King Packaged Materials Company, Burlington, Ontario, Canada) where the long axis of the tibia was parallel to the sides of the metal potting box.

### 2.2. Biomechanical Testing

**Figure 1:**
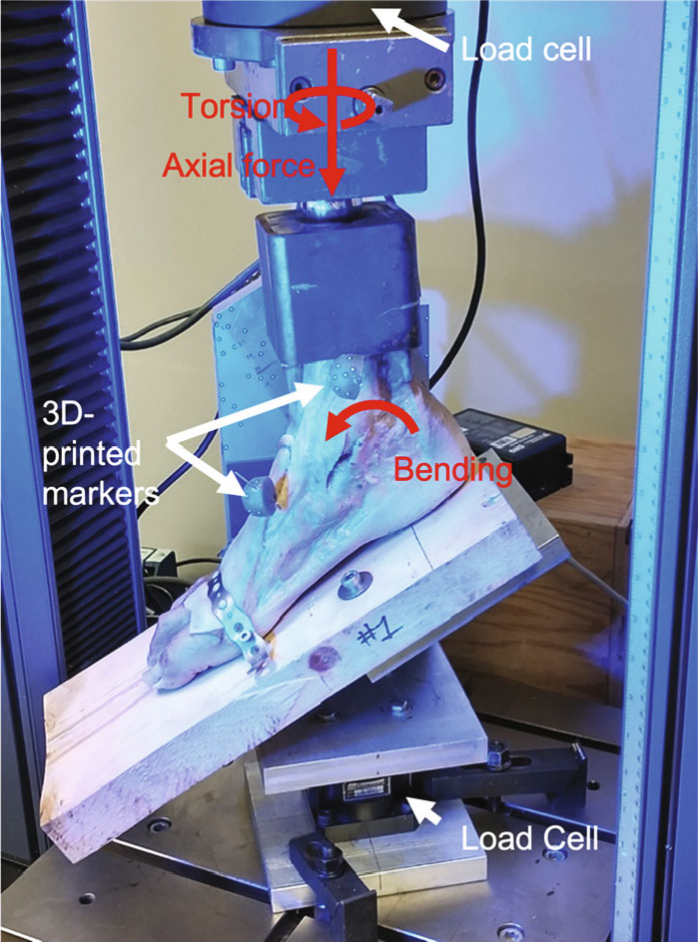
Mounted cadaveric specimen setup. The forefoot and hindfoot are attached rigidly to the footboard, and a consistent axial load stabilizes the construct within the dual-column test frame. Red arrows indicate the loading direction. White arrows show load cells and the attachment of the 3D-printed markers.

All biomechanical tests were performed at room temperature (20°C/68°F). Range of motion testing was conducted in three parts: plantarflexion/dorsiflexion (PF/DF), inversion/eversion (IV/EV), and internal rotation (IR)/ER. A custom-designed testing apparatus with a stepper motor, a 6-DOF load cell (MC3A, Advanced Mechanical Technology, Inc., Watertown, Massachusetts, United States), and a 5967 Dual Column test frame (Instron, Norwood, Massachusetts, United States) were used to apply pure moment loading (Figure 1). The forefoot was rigidly attached to a footboard via metal strapping across the midfoot, and the calcaneus was secured vertically with a screw. A metal ball Instron attachment rested in a smooth divot on the proximal end of the metal potting box allowing the ankle joint to move freely without restricting the tibia during PF/DF and IV/EV testing. For IR/ER, the metal potting box was rigidly attached to the Instron load cell via a vice. The neutral position was designated with the long axis of the tibia being perpendicular to the footboard. The testing rig's motor axis was aligned between the medial and lateral malleoli in the coronal plane and transverse plane and the center of the tibia in the sagittal plane.

An ARAMIS Adjustable 12M system (GOM Metrology, Braunschweig, Germany) was used as a digital imaging camera (DIC) system to track the motion in 6 degrees of freedom. The setup consisted of two 24-mm focal lengths and 4096-by-3600 pixels (pixel size of 3.45 *μ*m) resolution cameras. The two cameras were set at a 25° angle, 3 Hz, and illuminated with two polarized LED light sources. The DIC setup was calibrated prior to each biomechanical test by a standard calibration protocol and calibration plate provided by GOM Metrology for a measuring volume of 270-by-210-by-150 mm^3^. The images were processed in ARAMIS Professional 2019 (GOM Metrology, Braunschweig, Germany). At each step, the DIC setup captured the position of two 3D-printed markers inserted in the tibia and talar neck, and using rigid body kinematics, the ROM of the ankle was calculated. The unconstrained ankle joint was free to rotate/bend during the biomechanical tests. This allowed for a more natural ankle movement, which often leads to coupled motion, especially between IN/EV and IR/ER. Therefore, to separate the in-plane motion (motion in the direction of the motor rotation) and the out-of-plane motion (motion perpendicular to the motor rotation), the DIC's coordinate system was defined along the ankle joint and the motor axis of rotation. The in-plane motion was then used to calculate ROM and stiffness. The out-of-plane motion was highly specimen-specific, smaller, and deemed not useful to this study's results.

Specimens were loaded quasistatically with an axial preload of 15 N for mounting and to eliminate mechanical slack. Trial testing was performed to determine the degree of loading. PF/DF and IV/EV loading limits were set to 5.5 Nm, while ER/IR was set to 4 Nm to achieve complete ankle motion without damaging the specimens. Ankles were first inspected for deficiencies and tested intact as a control. Then, the deltoid was disrupted (sectioned) and tested. Lastly, the paired ankles underwent either repair or reconstruction chosen at random and tested. Left and right ankle pairs were matched with an equal number of repairs and reconstructions. In total, 12 ankles were tested intact and in the deficient state, 6 ankles were tested after reconstruction, and the other 6 ankles were tested following repair. Each test consisted of three cycles at 1°/s with the first two cycles being used as preconditioning to reach steady state and the last cycle utilized for data analysis. The center of mass of each ankle was calculated based on the center of rotation, and the resulting torque produced by the mass of the foot was removed from the calculations. Stiffness was approximated by taking the slope of the torque-angle graph between the minimum and maximum torque values. The ROM and stiffness results were normalized to the intact state.

### 2.3. Deltoid Defunctioning and Repair/Reconstruction Protocol

**Figure 2:**
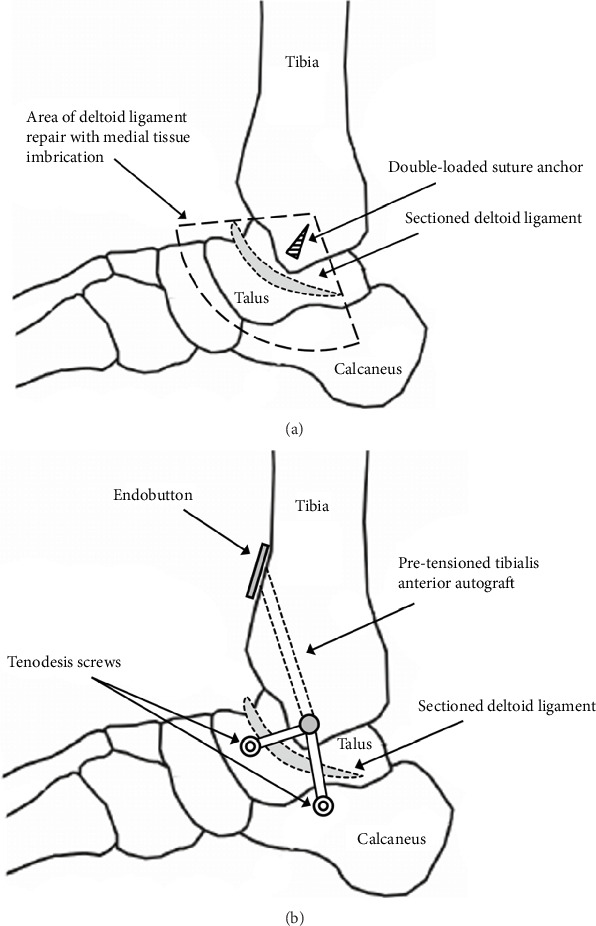
Schematic representation of deltoid ligament repair (a) and reconstruction (b).

Once the specimen was tested intact, the deltoid ligament was cut (sectioned) from the medial malleolar attachment, which is the most commonly seen deltoid ligament tear [19]. Once retested postdeltoid release, the ligament would either be repaired or reconstructed based on previous randomization. A standard double-loaded suture anchor was used for the deltoid repair. This involved identifying the distal remnants of both the superficial and deep deltoid components of the ligament. A suture anchor was inserted between the anterior and posterior colliculus, and the heavy nonabsorbable suture was used in a Mason–Allen configuration to imbricate tissues and reattach the deltoid ligament to its attachments on the medial malleolus (Figure 2a).

The TA was used as an autograft for the deltoid ligament reconstruction. The tendon of each specimen was freed proximally during initial preparation and potting. Before proceeding with testing, it was released distally from the medial cuneiform and first metatarsal base. The tendon was then prepared by removing any remaining muscle and performing a whipstitch at both ends. The tendons were pretensioned and wrapped in saline-soaked gauze. A modification of the deltoid ligament reconstruction technique outlined in Haddad et. al was used [20]. The deep anterior tibiotalar ligament footprint was identified on the talus, and a guidewire was passed medial on the footprint to lateral in the talar body, exiting anterior to the fibula. The footprint of the superficial tibiocalcaneal ligament was then identified on the calcaneus, and a second guidewire was passed from medial to lateral from the sustentaculum tali of the calcaneus. The intercollicular groove was then identified at the medial malleolus, and a third guidewire was inserted aiming at the anterolateral tibial cortex. The graft was folded to create a Y shape with asymmetric limb lengths. The graft size was measured; the tibial tunnel was drilled. The graft was affixed proximally in the anterolateral tibia via an endobutton. The graft was then sized at the shorter talar end, as well as on the longer calcaneal side. Talar and calcaneal tunnels were subsequently reamed 1 mm larger than the measured graft sizes. The ankle was placed in IV, and the graft ends were passed through the prereamed tunnels in the talus and calcaneus. Tension was applied and an appropriated sized tenodesis screw (5 or 6 mm in all specimens) was placed in the talus and sustentaculum tali of the calcaneus (Figure 2b).

### 2.4. Data Analysis

Statistical software R (version R-4.1.2) was used to determine any significant difference (*p* < 0.05). Shapiro–Wilk and Levene's tests were used to confirm normality and homogeneity of variance within the data. To account for the six matched pairs (12 total ankles from six donors), a linear mixed effect model was implemented where ROM and stiffness were the outcome variables, the deltoid ligament state (deficient, intact, repair, and reconstruction) was the fixed variable, and the matched pairs were grouped as random effects variables. A Tukey's Honest Significant Difference (Tukey HSD) method was used to identify all possible pairwise significant differences while keeping family-wise error rates low.

## 3. Results

### 3.1. Absolute Range of Motion and Stiffness

**Figure 3:**
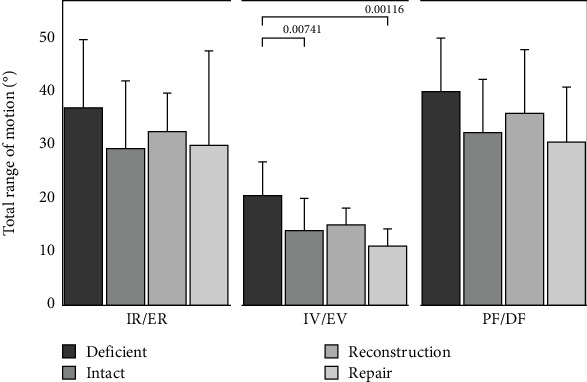
Total range of motion of specimens for deficient, intact, reconstruction, and repair states, in the three motion directions tested (IR/ER, IV/EV, and PF/DF, respectively). Statistical differences are denoted with lines and *p* values.

**Figure 4:**
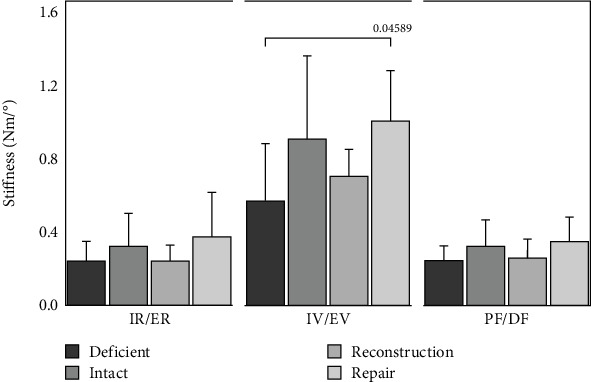
Stiffness (newton-meter/degree) of loading direction for deficient, intact, reconstruction, and repair states. Statistical differences are denoted with lines and *p* values.

The total ROM and stiffness values for each ankle can be seen in Figures 3 and 4, respectively. For all three loading directions (Figure 3), the deficient ankles on average had the most motion followed by the reconstruction than intact or repair, but significance was only detected in the IV/EV loading direction between the deficient and intact (*p* = 0.0074) and deficient to repair (*p* = 0.0012).  Figure 4 shows the stiffness of the ankle. The deficient ankles on average had the lowest stiffness followed by the reconstruction, intact, and lastly the repair on average was the stiffest. During IV/EV, the repair and deficient groups were significantly different (*p* = 0.0459) but no other significant difference was detected (*p* > 0.076). The numerical *p* values for ROM and stiffness both absolute and normalized to intact can be seen in Table 1.

### 3.2. Normalized Range of Motion

**Figure 5:**
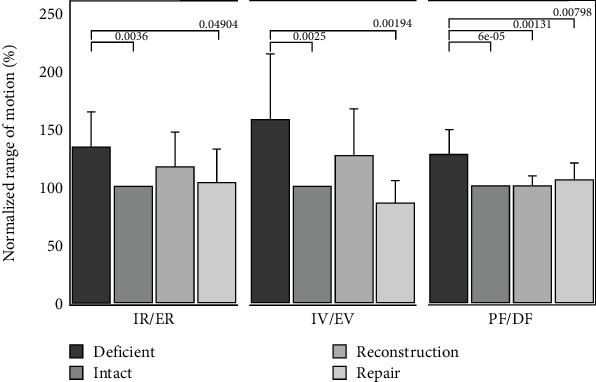
Normalized range of motion (ROM) for deficient, reconstruction, and repair states in each of the three primary axes tested (IR/ER, IV/EV, and PF/DF, respectively). Statistical differences are denoted with lines and *p* values.

Figure 5 indicates the normalized to intact ROM for the loading directions of IR/ER, IV/EV, and PF/DF, respectively. During PF/DF loading, the completely deficient ankle's normalized ROM (128.0% ± 21.5%) was significantly greater than the intact, reconstruction (101.1% ± 8.3%), and repair groups (105.6% ± 15.1%) with *p* = 0.0001, 0.0013, and 0.008, respectively. For IV/EV loading, the deficient ankle normalized ROM at 158.1% ± 56.8% was significantly greater than the intact (*p* = 0.0025) and repair state at 85.2% ± 20.2% (*p* = 0.0019) but was not significantly different from the reconstruction state at 126.6% ± 40.4% (*p* = 0.31). For IR/ER loading, the deficient ankle was only significantly different from the intact state at 134.3% ± 30.7% (*p* = 0.0036) and the repair state at 103.9% ± 28.6% (*p* = 0.049). For all the experiments, the intact, reconstruction, and repair states normalized ROM were never significantly different (*p* = 0.21–0.99).

### 3.3. Normalized Stiffness

With regard to normalized stiffness, the deficient ankles were significantly different at 77.8% ± 13.4% to the intact (*p* < 0.0001), the reconstruction states at 99.4% ± 7.9% (*p* = 0.0006), and the repair state at 95.9% ± 18.0% (*p* = 0.0045) during PF/DF loading. For IV/EV loading, the deficient ankle was significantly different at 66.9% ± 16.4% than the intact state (*p* = 0.0016) and repair state at 122.6% ± 29.4% (*p* < 0.0001) but not to the reconstruction state at 86.6% ± 31.8% (*p* = 0.21). However, the repair state was significantly stiffer than the reconstruction state (*p* = 0.018) and was trending to be stiffer than the intact state at *p* = 0.12. Lastly, in IR/ER loading direction tests, the deficient state was significantly less stiff compared to the intact state at 78.2% ± 19.2% (*p* = 0.028) and the repair state at 102.8% ± 31.4% (*p* = 0.049) while the intact to repair to reconstruction was not significantly different to each other (*p* = 0.53–0.98). Reconstruction and repair states were significantly similar to the intact state in all loading directions.

## 4. Discussion

This biomechanical study sought to assess differences in stability of cadaveric ankle specimens with intact, deficient, repaired, or reconstructed deltoid ligaments. When specimens were normalized to their intact ROM, we found that there was no significant difference between repair, reconstruction, and intact states across all axes tested (IV/EV, PF/DF, IR/ER). Importantly, our study found that sectioning of the deltoid ligament generally decreased ankle stability and increased the motion seen in the ankles, while reconstruction and repair led to a reduction in motion and ankle stabilization. Overall, the normalized data for the deficient ankles differed from the intact and repair states while no significant difference between intact/repair/reconstruction states was seen. Therefore, reconstruction or repair was able to stabilize the ankle joint reducing the motion seen to be closer to the intact state. These findings suggest that in the event of a deltoid ligament rupture, both repair and reconstruction of this ligament confer additional stability to the ankle joint. This study highlights a direct biomechanical comparison between deltoid reconstruction and repair and suggests that, when feasible, ligament repair generally confers additional stability in comparison to reconstruction.

The deltoid ligament is thought to be responsible for limiting hindfoot EV (superficial) and ER (deep); a reconstructive design must therefore adequately achieve both functions. A previous biomechanical study by Haddad et al. [20] suggested that reconstruction of the deltoid ligament is effective in achieving these limitations in motion. After normalizing the angular ROM/stiffness data, they found the reconstruction state was significantly stiffer than the deficient state in EV and ER (at 89.8% and 136% compared to the intact group) but not in IV or IR. In comparison, our results show no significant difference in normalized ROM for the reconstruction state versus any other states in IR/ER or IV/EV but a lower average normalized ROM than the deficient state. Additionally, Haddad et al. reported no significant difference in normalized ROM and normalized stiffness in PF/DF loading while we found a significant difference between the deficient state and all other states during PF/DF loading. They observed that sectioning the deltoid increased IV and EV by 10° and combined ER and IR by 15° at 2 Nm torque, and reconstruction reduced the apparent motion by a combined 9.5° and 10° for IV/EV and IR/ER, respectively. Similarly, we found on average a 6.5° and 8.5° increase after sectioning the deltoid for total IV/EV and IR/ER motion, respectively; reconstruction reduced the motion by 5.5° and 5.3° and repair reduced the motion by 9.4° and 8° for IV/EV and IR/ER, respectively. A similar biomechanical study found a significant increase (*p* < 0.01) of 10.5° on average in combined ER and IR after disrupting both the deltoid and syndesmosis; after repairing the deltoid alone, the rotational ROM decreased on average by 4.1° but was not significantly different from the intact or deficient state [21]. Differences in published results can be partially accounted for by the difference in the test setup and the torque limit set. Our biomechanical testing setup utilized a 5.5 and 4 Nm limit to ensure a full ROM for the intact ankle, but physiological limits at which the ankle's rotation is impeded by other biological factors limit the potential difference that can be seen between the deltoid ligament states. Limiting the amount of motion in the intact state may highlight the deltoid's impact but is not indicative of the full ROM seen in the ankle nor the strength of the fixation at normal physiological ankle torques.

Surgical versus nonsurgical treatment of deltoid ligament ruptures and potential subsequent instability remains controversial. For a better analysis of this topic, it is necessary to divide deltoid ligament deficiencies into acute and chronic cases. Acutely, it has been suggested through arthroscopic examination that up to 40% of ankle fractures include a deltoid injury [22]. In addition to physical exam findings of ecchymosis and medial tenderness, a DF–ER stress view is most commonly used by surgeons to diagnose deltoid injuries with magnetic resonance imaging (MRI) and ultrasound forming other imaging options [23]. Stress fluoroscopy is used intraoperatively to assess the integrity of the repair. Weight-bearing radiographs at 3 months postoperatively have been associated with decreased medial clear space in bimalleolar equivalent fractures following deltoid ligament repair [24]. Our study complements this data by showing an overall decrease in ROM and an increase in stability following repair/reconstruction of the sectioned ligament. Part of the challenge in assessing the efficacy of deltoid reconstruction/repair compared to nonsurgical treatment is correlating these improved radiographic and cadaveric outcomes (appropriate medial clear space) with functional outcomes [9, 25]. It is reasonable to speculate that an increased medial clear space will lead to altered biomechanics and instability with subsequent development of posttraumatic arthritis. If repair/reconstruction of the deltoid complex is critical to achieving a favorable radiographic outcome, improved functional outcomes should follow with longitudinal study [26, 27].

There have been several cases of chronic medial ankle instability after remote injury described in the literature [28, 29]. These patients would ultimately present with chronic deltoid ligament insufficiency. This finding is also seen in patients with Stage IV posterior tibial tendon insufficiency. In these patient populations, repair is often not possible and several reconstruction techniques using auto- or allograft have been described [20, 30–32]. In posterior tibial tendon insufficiency, deltoid ligament reconstruction has been described both as a precursor to ankle arthroplasty and as a standalone salvage procedure [31, 32]. In the context of ankle arthroplasty, coronal plane deformity of more than 15° has been associated with edge loading and increased implant wear [33, 34]. The goal of deltoid ligament reconstruction in these cases is to reinstate medial ankle stability to allow for better anatomic positioning and durability of ankle prostheses. Clinically, good radiographic and functional results have been reported in a patient population who underwent deltoid ligament reconstruction without ankle arthroplasty [32]. This study provides additional biomechanic evidence that reconstruction can indeed be an effective tool in restoring ankle biomechanics closer to its intact state.

There exist limitations to this study. Given that this is a cadaveric biomechanical study, the results can only be extrapolated to the early postoperative period prior to stabilization conferred by healing and additional scarring. Therefore, limited long-term clinical conclusions can be drawn from this data. In addition, inherent interspecimen variations exist between different cadaver pairs and within each pair which we attempted to alleviate in part by normalizing the data to its intact state. However, the repeated use and the semirandomized testing order may have led to unintended damage of the specimen at the level of torque implemented compromising the integrity of the ankles for subsequent tests. For consistency between groups, the deltoid was sectioned completely, but this does not account for the myriad of potential injuries seen clinically and may therefore not be fully generalizable. A recent biomechanical study highlights the difference in the instability pattern caused by sectioning various parts of the deltoid ligament complex [7]. The differences in results to similar published biomechanical experiments may in part be accounted for by the difference in the experimental setups including torque limits, loading rates, preloads, and methods to mount the specimen for pure moment loading. Little difference in the results was seen when isolating true EV and ER from IV and IR, and therefore, the data was analyzed in the complete ROM arc for each plane to reduce any errors generated when mounting the specimen. Future biomechanical tests could highlight potential differences between reconstruction and repair of the deltoid such as failure loading, fatigue testing, shear loading, and gait analysis to further elucidate the advantages and disadvantages between the two techniques. Lastly, with six matched pairs, the analysis may have been underpowered to differentiate all possible clinical differences, as a post hoc power analysis (at 80% power) indicated a sample size of 473 would be needed to determine the statistical difference for normalized ROM between all states, which is not feasible. An increased sample size may mitigate some of the listed limitations (i.e., residual errors from specimen-specific effects being reduced with increased sample size) and further elucidate potential differences between intact, repair, and reconstructed states.

## 5. Conclusion

In conclusion, this study highlights that deltoid repair and reconstruction are capable at time zero of decreasing instability caused by sectioning of the deltoid ligament. These results suggest that in cases where chronic deltoid ligament insufficiency and instability repair is not feasible, reconstruction may be used as a reliable alternative; future studies examining additional biomechanical parameters and further clinical trials to see any difference in healing and efficacy are warranted.

## Figures and Tables

**Table 1:** Range of motion (ROM), normalized to intact ROM, stiffness, and normalized to intact *p* values, where ^N^ denotes normalized to intact.

**Comparison**	**ROM**	**ROM** ^**N**^	**Stiffness**	**Stiffness** ^**N**^
IV/EV				
Intact–deficient	0.0074⁣^∗^	0.0025⁣^∗^	0.0767	0.0016⁣^∗^
Reconstruction–deficient	0.114	0.3143	0.8728	0.2082
Repair–deficient	0.0012⁣^∗^	0.0019⁣^∗^	0.0459⁣^∗^	< 0.0001⁣^∗^
Reconstruction–intact	0.9636	0.458	0.6003	0.5258
Repair–intact	0.548	0.8407	0.9114	0.1201
Repair–reconstruction	0.4341	0.2106	0.3674	0.0176⁣^∗^
PF/DF				
Intact–deficient	0.1127	0.0001⁣^∗^	0.1127	< 0.0001⁣^∗^
Reconstruction–deficient	0.6842	0.0013⁣^∗^	0.9285	0.0006⁣^∗^
Repair–deficient	0.1262	0.008⁣^∗^	0.0797	0.0045⁣^∗^
Reconstruction–intact	0.8795	0.9982	0.6039	0.9994
Repair–intact	0.9879	0.8174	0.9429	0.8257
Repair–reconstruction	0.7921	0.9273	0.4156	0.9165
IR/ER				
Intact–deficient	0.0631	0.0036⁣^∗^	0.2702	0.0277⁣^∗^
Reconstruction–deficient	0.5645	0.4393	0.9994	0.5316
Repair–deficient	0.207	0.049⁣^∗^	0.0858	0.0488⁣^∗^
Reconstruction–intact	0.8787	0.4168	0.5603	0.7214
Repair–intact	0.9981	0.9842	0.7878	0.9894
Repair–reconstruction	0.9551	0.7259	0.2374	0.6449

⁣^∗^Significant differences (*p* < 0.05).

## Data Availability

Raw data will be made available by the corresponding author upon reasonable request.
